# NIDM-Terms: community-based terminology management for improved neuroimaging dataset descriptions and query

**DOI:** 10.3389/fninf.2023.1174156

**Published:** 2023-07-18

**Authors:** Nazek Queder, Vivian B. Tien, Sanu Ann Abraham, Sebastian Georg Wenzel Urchs, Karl G. Helmer, Derek Chaplin, Theo G. M. van Erp, David N. Kennedy, Jean-Baptiste Poline, Jeffrey S. Grethe, Satrajit S. Ghosh, David B. Keator

**Affiliations:** ^1^Department of Psychiatry and Human Behavior, School of Medicine, University of California, Irvine, Irvine, CA, United States; ^2^Department of Neurobiology and Behavior and Center for the Neurobiology of Learning and Memory, University of California, Irvine, Irvine, CA, United States; ^3^Fairmont Preparatory Academy, Anaheim, CA, United States; ^4^McGovern Institute for Brain Research, Massachusetts Institute of Technology, Cambridge, MA, United States; ^5^NeuroDataScience–ORIGAMI Laboratory, McConnell Brain Imaging Centre, The Neuro (Montreal Neurological Institute-Hospital), Faculty of Medicine, McGill University, Montreal, QC, Canada; ^6^Massachusetts General Hospital, Boston, MA, United States; ^7^Harvard Medical School, Boston, MA, United States; ^8^Clinical Translational Neuroscience Laboratory, Department of Psychiatry and Human Behavior, School of Medicine, University of California, Irvine, Irvine, CA, United States; ^9^Center for the Neurobiology of Learning and Memory, University of California, Irvine, Irvine, CA, United States; ^10^Departments of Psychiatry and Radiology, University of Massachusetts Chan Medical School, Worcester, MA, United States; ^11^Department of Neurosciences, School of Medicine, University of California, San Diego, San Diego, CA, United States; ^12^McGovern Institute for Brain Research, Massachusetts Institute of Technology, Cambridge, MA, United States

**Keywords:** neuroimaging, dataset, query, annotation, NIDM

## Abstract

The biomedical research community is motivated to share and reuse data from studies and projects by funding agencies and publishers. Effectively combining and reusing neuroimaging data from publicly available datasets, requires the capability to query across datasets in order to identify cohorts that match both neuroimaging and clinical/behavioral data criteria. Critical barriers to operationalizing such queries include, in part, the broad use of undefined study variables with limited or no annotations that make it difficult to understand the data available without significant interaction with the original authors. Using the Brain Imaging Data Structure (BIDS) to organize neuroimaging data has made querying across studies for specific image types possible at scale. However, in BIDS, beyond file naming and tightly controlled imaging directory structures, there are very few constraints on ancillary variable naming/meaning or experiment-specific metadata. In this work, we present NIDM-Terms, a set of user-friendly terminology management tools and associated software to better manage individual lab terminologies and help with annotating BIDS datasets. Using these tools to annotate BIDS data with a Neuroimaging Data Model (NIDM) semantic web representation, enables queries across datasets to identify cohorts with specific neuroimaging and clinical/behavioral measurements. This manuscript describes the overall informatics structures and demonstrates the use of tools to annotate BIDS datasets to perform integrated cross-cohort queries.

## 1. Introduction

There is a “crisis of replication” in neuroscience (Button et al., 2013; Szucs and Ioannidis, 2017). Interpreting, reproducing, and validating results of experiments depends critically on our ability to understand the conditions under which the data were acquired and processed. Efficient discovery and reuse of existing data relies on the data and metadata adhering to the FAIR: Findable, Accessible, Interoperable and Reusable principles (Wilkinson et al., 2016; Schulz, 2018). The biomedical research community is motivated to share and reuse data from studies and projects by an increasing number of requirements from funding agencies (e.g., NIH-wide Policy for Data Management and Sharing^1^) and publishers (PMID: 34914921). There are a growing number of data repositories (Das et al., 2012; Book et al., 2013; Poldrack et al., 2013; Ambite et al., 2015; Crawford et al., 2016; Kennedy et al., 2016), each with their own data structures and data dictionaries (Eickhoff et al., 2016). With dozens of neuroimaging data sharing sources now available, we need better methods to annotate datasets and to search across those datasets without a significant investment in time to develop database mediation services (Keator et al., 2008; Turner et al., 2015; Wang et al., 2016; Niso et al., 2022) or creating “crosswalks” mapping variables across datasets.

Critical barriers to finding and reusing data include the use of undefined variables and/or an insufficient degree of variable annotations that make it difficult to understand the data available without significant interaction with the original authors. Further, determining whether cohorts from different studies can be combined, based on phenotypes or acquisition parameters is currently difficult, requiring a significant investment in effort from the researcher. The ability to conduct searches across diverse datasets is difficult and typically requires sufficient annotation of the study variables to understand what was collected and how to query each dataset to find meaningful results. For example, a query such as: “identify datasets that contain a measure of depression, age, IQ, and a T1-weighted MRI scan” is not easy to implement. Historically, this type of query would have to be posed to multiple data repositories separately, through each repository’s interface, and the results manually combined by the investigator. Often, the returned results would depend upon the annotations used in each repository and the level of granularity to which each data object was annotated which may require the investigator to download complete datasets in order to manually extract the data of interest. In some cases, this query can not be satisfied without an expert user because often the same annotation term collection is not used across repositories, as terms used to annotate collected study variables are inconsistent. Each lab can freely name study variables such that they are not guaranteed to be meaningful or sufficient, either for understanding or for querying each interface and each dataset.

Building off the example query above, it has proven difficult to query arbitrary datasets to find out whether they contain images with contrast types relevant to the research question. The Brain Imaging Data Structure (BIDS) (Gorgolewski et al., 2015; incf-nidash, 2016) was designed to provide software developers and the neuroimaging community with file- and directory-naming conventions for organizing imaging data. Because of its simplicity, BIDS has been quickly supported by a number of analysis tools and database platforms (COINS,^2^ XNAT,^3^ Scientific Transparency,^4^ OpenfMRI,^5^ LORIS^6^). In BIDS, the organization of the data is required to conform to strict naming and directory-structure conventions. The adoption of BIDS has addressed the imaging-related parts in our example query above because with BIDS and the associated PyBIDS^7^ Python library, one can use the location of data within the directory structure to determine the type of images included in that dataset. Beyond file naming and tightly controlled imaging directory structures, there are very few constraints on ancillary variable naming/meaning or experiment-specific metadata in BIDS. As such, we still have difficulty satisfying the query above because: (1) we cannot guarantee that variable names will be meaningful; (2) data dictionaries are optional in BIDS and there is no validation that data dictionaries, if supplied, contain important or sufficient information (e.g., units, frames of reference, etc.). Therefore, searching and combining information across independent BIDS datasets is often difficult for data beyond image types and metrics describing those images. Finally, there is no query engine that natively supports BIDS datasets.

To address these concerns regarding the ability to query across BIDS datasets, as well as the desire to create a web of linked human neuroimaging data, an international team of cognitive scientists, computer scientists, and statisticians are developing a (meta)data representation model and tools to support its use. The goal is to provide the foundational infrastructure in a well-defined and easily expandable model, to link datasets using unambiguous annotations. This effort, built upon the resource description framework (RDF) and the PROV standard^8^ (Moreau et al., 2008; PROV-Overview, 2016), is called the Neuroimaging Data Model (NIDM)^9^ (Keator et al., 2013; Maumet et al., 2016; NIDM, 2016). By using RDF as the foundation for NIDM, it benefits from a variety of sophisticated query languages (e.g., SPARQL, RQL, TRIPLE, Xcerpt), an open world assumption allowing users to add as many statements about the data as they like without constraints on header sizes as is the case with typical image formats, and direct use of web-accessible terminologies and ontologies to provide multiple layers to link and infer relationships among data and metadata. A full description of NIDM is beyond the scope of this manuscript, but NIDM was designed to facilitate queries across neuroscientific datasets. A Python library was built (PyNIDM^10^) to create NIDM annotation documents and a tool was also created to represent a BIDS dataset, along with all the associated behavioral and/or clinical data, as a NIDM document. Using PyNIDM and NIDM documents, one could use RDF query languages to satisfy the example query above across BIDS or other datasets.

To annotate datasets for future discovery or integration, researchers need to be able to rely on a set of common properties for precisely defining study variables, beyond what is already offered by BIDS for imaging data. In other domains beyond neuroimaging, tools have been developed to aid in dataset annotation such as the open source ISA framework (Sansone et al., 2012) for life sciences research, the Clinical Data Interchange Standards Consortium (CDISC) RDF framework (Facile et al., 2022) focused on the medical and healthcare domains, and Frictionless Data^11^ developed to support climate scientists, to humanities researchers, to government data centers, and others. In this manuscript, we focus on the research neuroimaging community yet many of the methods presented are general and could be applied to other domains in synergy with related efforts. Here we describe NIDM-Terms, a toolkit that employs both the NIDM data model and associated terminologies to aid in querying across datasets. We provide tools to more fully annotate BIDS datasets and provide user-friendly community-based annotation and terminology management tools to assure proper definitions and metadata are provided with the annotations. Further, we show how these annotations, along with the NIDM data model, can be used to search across publicly available neuroimaging datasets.

## 2. Materials and methods

In the following sections, we begin by formalizing our definitions of different data element types. We then define a small set of properties we consider critical to include when annotating study-specific data elements to be able to both understand, at a high level, what was collected and assure such annotations have the necessary information for researchers to understand how to reuse and/or combine these with other studies. Finally, we describe some tools, both command-line and graphical, for creating these data element annotations.

### 2.1. Data element types

Data elements can be simply defined as annotations on data, where data can be variable names or content, file names or content of files. In this work, we introduce two distinct types of data elements and a conceptual element: (1) data elements that are often locally defined and represent study variables (personal data elements; PDEs), (2) data elements that are defined by a community or a standards body (common data elements; CDEs), and (3) terms that capture an abstract idea or a general notion (concepts). Within the NIDM terminology work, each of these distinct types of elements play an important role in the detailed description of datasets.

Personal Data Elements (PDEs) refer to the typical study variables and require strict definitions, ranges, value types, and, if categorical, complete definitions of the categories and their potential mapping from numerical categories to text-based strings (e.g., 0 = right handed, 1 = left handed, 2 = ambidextrous) to be easily reused across studies. PDEs may define common terms in non-standard ways or use non-standard terms for commonly acquired variables or processing steps. PDEs may also combine separate annotation terms into a single term, e.g., “age_months” that combines the duration “age” with the units of “months.” In general, since PDEs are used locally, there is no requirement to adhere to a standard convention and users are typically free to name and annotate such elements as they wish.

Common data elements (CDEs) are those that have been adopted for use by a group, often either a consortium operating in a specific domain or standards body. Ideally, a rigorous adoption process is implemented that entails the proposal of a term, identification of whether similar terms already exist in other terminologies, determination of how the term will fit into the logical structure of the existing terminology and whether it adheres to standards already established by the group. Often though, CDE collections may be simply that, a collection of terms that a group has decided to use, without the establishment of any standards or logical framework.

Concepts are distinctly different from CDEs and PDEs. Concepts (also known as “classes” in RDF) are those terms that represent “higher order” ideas, e.g., the concept of “age” is the notion of a duration of time from some predetermined starting point to the current moment. Concepts are used to aid in querying across datasets and provide a mechanism for researchers to annotate their study-specific PDEs (or CDEs if they are used within a study) with abstract ideas or general notions about a PDE which helps us to query across datasets. For example, two studies collect a data element meant to measure the participant’s dominant hand. Dataset one names the variable simply “handedness” and is stored as a categorical variable with values indicating whether the participant is predominantly right-handed, left-handed, or ambidextrous. Dataset two instead collects the Edinburgh handedness inventory, names their study variable “ehi” and whose values are integers ranging from −40 (left handed) to 40 (right handed). Therefore, *no query for a single variable name would return data from both datasets*. However, if each dataset annotated their handedness assessment data with a concept describing the general notion of “handedness assessment,” for example term *ILX:0104886*^12^ in the InterLex repository, querying across datasets would then return handedness data from both datasets. One could then investigate each returned dataset and understand, through the data element properties (see section “2.2. Properties”), the distinctions between how each was measured.

The use of properly defined CDEs, concepts, and properties provides the foundation for NIDM documents to: (1) abstract the concepts inherent in PDEs to allow for meaningful searches across data collections, (2) provide an extensible collection of general and domain-specific terms used to describe data, and (3) allow for an inherently flexible annotation of data to an arbitrary level of detail. As an example of the above points, reconceptualizing the PDEs “age_months” and “YEARSOLD” from different datasets with the properties “isAbout” the concept “age” and “hasUnits” of “months” and “years,” respectively, allows an automated system to discover both PDEs when “age” is searched for, as well as not having to define a separate variable each time a different duration unit is required.

### 2.2. Properties

Properties play an important role in disambiguating and simplifying the annotation of data, as well as the mapping of data elements between data sources, especially those that use PDEs. In reviewing available terminologies and ontologies for use in human neuroimaging studies, we found that data elements in these terminologies often lacked important properties such as units, value types, ranges, etc. When researchers try to reuse data collected by other laboratories they often request data dictionaries which describe the study variables collected (PDEs) and hopefully provide precise definitions and properties for those variables. If important properties are missing, such as “units,” the data is either not usable or users must contact the dataset providers, if they are reachable, to correctly and confidently reuse the data. The NIDM team found this to be a significant problem when trying to reuse retrospective data and query across studies to build cohorts matching various search criteria. We therefore started out by defining a minimal set of properties (Table 1) that we felt were important to properly define data elements of various types (see section “2.1. Data element types”). A larger set of properties are available in the terminology used in the experimental description component of NIDM (i.e., NIDM-Experiment).

**TABLE 1:** Data element properties.

Property	Definition
Description	An explanation of the nature, scope, or meaning of the data element.
Label	Short text string for referring to the data element.
ValueType	A value representation such as integer, float, string, date/time (e.g., xsd: int, xsd: float, xsd: string).
UnitCode	Unit of measurement (e.g., years, millimeters, etc.).
MaxValue	The upper value of the data element (in case of ordered data).
MinValue	The lower value of the data element (in case ordered data).
Choices	Choices is a concept that corresponds to the BIDS (https://bids.neuroimaging.io/) “levels” standard for categorical variables where you’re mapping the value (often an integer) to some text string. Using the handedness example from above, the choices would be {1 = Right, 5 = Left, 10 = Ambidextrous}.
IsAbout	Used to record the relationship between a data element and a broader concept. Annotating using is About can be used to search across datasets. The is About annotations consist of a url to identify the concept and an optional label for the concept.
Source_variable	Variable name from dataset. This applies to personal data elements which are data elements defined within a specific study, typically referred to as “study variables.”
MeasureOf	Describes what the data element measures (e.g., volume, area, distance, intensity, health status, duration/period, intelligence).
datumType	What type of datum it is (e.g., range, count, scalar etc.).
IsPartOf	Used to link data elements to assessments (e.g., WAIS_Vocab_Raw linked to WAIS scale (https://www.cognitiveatlas.org/task/id/tsk_4a57abb949f12/#). Typically this is not added by the user and is often done as an additional annotation to link data elements with other classes of information.
SubtypeCDEs	This property is typically added during curation. It links the term to lower-level (child) terms to provide some limited ontological relationships.
SupertypeCDEs	This property is typically added during term curation. It links the term to higher-level (parent) terms to provide some limited ontological relationships.
AssociatedWith	List of strings used to associate data elements with communities (e.g., BIDS, NIDM, etc.) for grouping data elements or searching within communities for specific data elements.

Many of the terms that are used for annotation are part of NIDM-Experiment (NIDM-E), an ontology that can be used to describe neuroscience experiments. NIDM-E was originally constructed with an emphasis on terms describing imaging-based studies, in particular those employing MRI, but has since been expanded to encompass other modalities. NIDM-E was built through the annotation of several real-world multi-modality neuroscience-based data sets. The goal of NIDM-E is to provide semantically-aware tools, a collection of defined terms organized in a structure that can be used to annotate data to an arbitrary level of detail. The structure of NIDM-E allows a user both to annotate complicated data collections and accommodate terms for new modalities and acquisition methods. NIDM-E also comprises tools to discover terms, webpages for term URL resolution, and a framework for community conversations regarding the terms.

As per good ontological practice (Arp et al., 2015), NIDM-E reuses terms from other ontologies before creating new terms. Terms are reused from such active ontologies such as the Semanticscience Integrated Ontology (SIO) (Dumontier et al., 2014), Information Artifact Ontology (IAO) (Ceusters, 2012), and Prov-O.^13^ These general ontologies provide the framework to which domain-specific terms were added to create NIDM-E. Terms created for NIDM-E have formal Aristotelian definitions in the “X is a Y that Z” format (Seppälä et al., 2017). NIDM-E also includes many imported data type, object, and annotation properties.

Because NIDM-E began with neuroimaging data, it has particularly strong coverage in that domain. It contains two unique properties: “hadImageContrastType” and “hadImageUsageType” that can be used to distinguish between the physics-based mechanism for the contrast in an image (e.g., “T2-weighted”) and the eventual use of that image (e.g., “Anatomical”). These are important for the discovery of imaging data in and across repositories, where datasets with different image contrasts may be annotated by the same term. For example, T2*-weighted, and T2-weighted images may both be stored as “Functional” data. NIDM-E also includes terms from two widely used standards: DICOM^14^ and the BIDS standards, the former of which is ubiquitous in the neuroimaging domain for the formatting of raw image data and is used in multiple imaging modalities. We have created a set of DICOM tag data type properties that can be used to associate acquisition parameters with an acquisition object. We have also included BIDS terms so that BIDS-organized datasets can be annotated using terms directly from the official BIDS schema.

**FIGURE 1:**
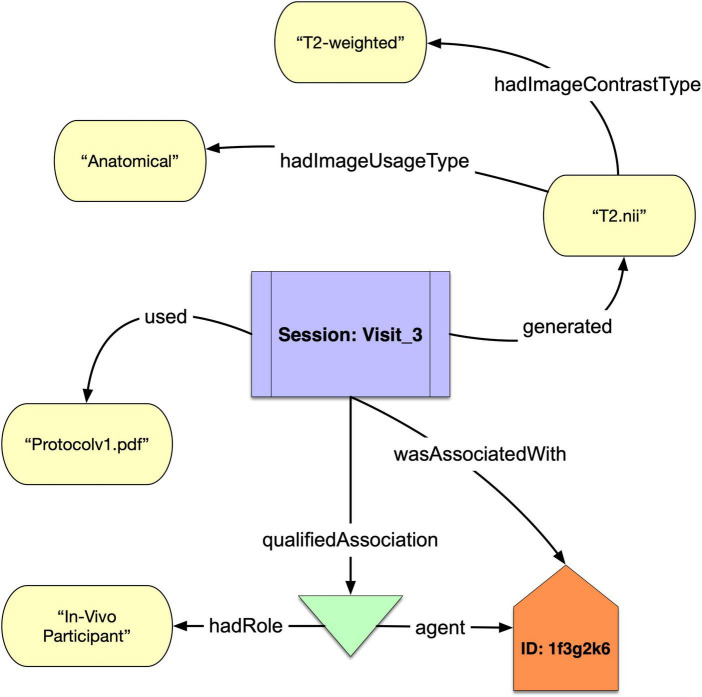
A schematic of a acquisition object “T2.nii” that was generated from the Session “Visit_3” of participant “1f3g2k6” annotated with the protocol used and “hadImageUsageType” and “hadImageContrastType.”

We show in Figure 1 a simple example of how NIDM-E can annotate an acquisition object, “T2.nii,” with an image contrast type of “T2-weighted” and an image usage type of “Anatomical,” and showing the scan session activity it was acquired at (“Session:Visit_3”), the protocol that was used (“Protocolv1.pdf”), and the study participant (“ID:1f3g2k6”) from which it was acquired and who had the role of “*In Vivo* Participant.”

The NIDM-E term-resolution and schema pages are available in GitHub^15^ which includes a web-accessible infrastructure built so that (1) the neuroscientific community can suggest or edit terms to the NIDM-E vocabulary using GitHub issue templates, (2) terms have resolvable URI’s, and (3) the ontology can be browsed to facilitate term discovery. GitHub issue templates allow us to have a public record of the discussion surrounding each term. To discover NIDM-E terms, we provide a “Schema Browser”^16^ webpage that allows users to view the NIDM-E term graph, including all of the imported terms. For semantic-web applications, we also provide a “Terms Resolution”^17^ page in which each term has a unique URL so that terms used by applications have a unique reference location.

### 2.3. SHACL validation

Beyond just defining useful properties for annotating data elements, it is critically important that researchers include such properties in their data annotations (i.e., data dictionaries). To ensure that data elements annotated by the community and contributed to the NIDM-Terms ecosystem contain the appropriate properties according to their type, we have built a validation schema using the Shapes Constraint Language (SHACL) (Pareti et al., 2019). SHACL is a W3C-supported language for validating RDF graphs according to a schema (i.e., a SHACL shape). Each data element type (e.g., PDE, CDE, and concept) has a separate SHACL shape used for validation. These shapes specify the required properties, the value type of each property’s values, and the number of such properties in each data element definition. Validation is done when new data elements are added to the NIDM-Terms GitHub repository through pull requests, either using the NIDM-Terms UI (see section “3.1. NIDM-Terms user interface”) or through Github Actions and Github Pull Requests. The git action uses the Python validation framework provided by ReproSchema.^18^ In brief, Reproschema offers a way to standardize the underlying representation of assessment tools. It comes with an open and accessible library of questionnaires with appropriate conversion [e.g., from/to RedCap (Patridge and Bardyn, 2018)] and data collection tools [e.g., MindLogger (Klein et al., 2021), RedCap, etc.] to enable a more consistent acquisition across projects, with data being harmonized by design. The techniques described here have been aligned with ReproSchemas to both support automated annotation of data collected and shared using assessments from ReproSchemas and to align our data element descriptions so there is consistency across representations. Such consistency will help the user who wants to share their study data when collected using ReproSchemas.

### 2.4. Terminology management resources used in NIDM terms

In order to provide users with the ability to easily annotate their data and link selected PDEs to broader concepts, a simple means to query across existing terminologies is needed. These query services are provided by Interlex^19^ (Surles-Zeigler et al., 2021), a dynamic lexicon, initially built on the foundation of NeuroLex (PMID: 24009581), of biomedical terms and common data elements designed to help improve the way that biomedical scientists communicate about their data, so that information systems can find data more easily and provide more powerful means of integrating data across distributed resources. One of the challenges for data integration and FAIR data is the inconsistent use of terminology and data elements. InterLex allows for the association of data fields and data values to common data elements and terminologies, enabling the crowdsourcing of data-terminology mappings within and across communities. InterLex also provides a stable layer on top of the many other existing terminologies, lexicons, ontologies (i.e., provides a way to federate ontologies for data applications), and common data element collections to enable more efficient search for users. To support annotation using CDEs, InterLex has been expanded to include the full NIMH Data Archive (NDA) CDE library. Through available RESTful web-services, InterLex is supporting alignment of data elements and terminologies through PyNIDM developed to simplify creation, editing, and querying of NIDM documents. To further expand our available terminologies, PyNIDM supports querying the Cognitive Atlas^20^ as an additional information source for dataset annotation. Similar to Interlex, Cognitive Atlas provides a systematic approach to representing cognitive neuroscience entities and biomedical terminologies.

## 3. Results

In previous sections, we have described the foundational principles used in this work to annotate study variables. Research laboratories often reuse PDEs across research projects or, alternatively, define new PDEs for studies that have previously been used in other projects. In an effort to help labs maintain an internal list of PDEs and share them with others in the community, we have developed both terminology management and dataset annotation tools. In the following sections, we describe three such annotation tools and a terminology management interface. We then show how proper dataset annotations can be useful in querying across publicly available MRI-related neuroimaging data.

### 3.1. NIDM-Terms user interface

To facilitate the community’s interaction in managing the neuroimaging terminology, we developed a JavaScript (using Visual Code Studio: version 1.67.1) NIDM-Terms User Interface^21^ (UI), hosted on GitHub Pages, that allows community curators to define and interact with their lab-specific terminologies as well as reuse terms from other neuroimaging communities. The UI is designed around the Git version control system and uses the NIDM-Terms/terms^22^ GitHub repository as a backend, providing JavaScript Object Notation - Linked Data (JSON-LD)^23^ formatted files for each PDE, CDE, and concept contributed by the community.

**FIGURE 2:**
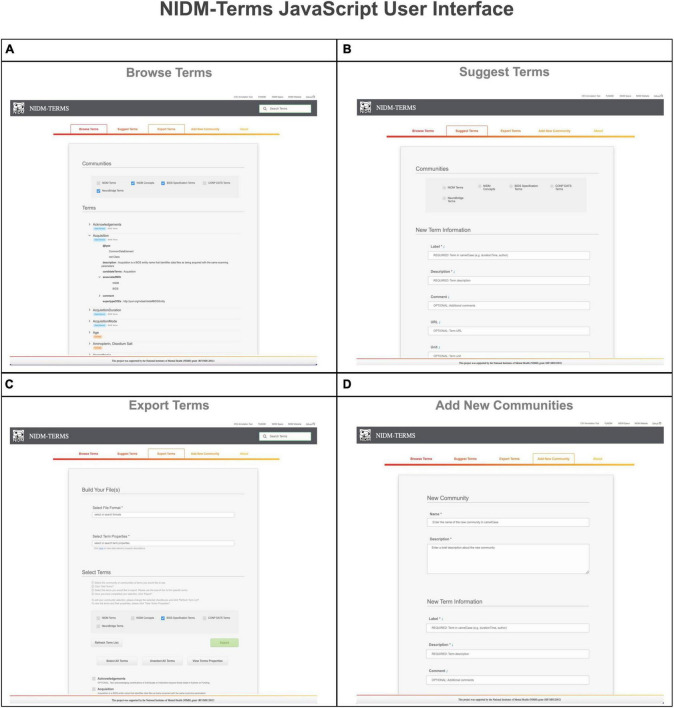
This figure illustrates the various functionalities the NIDM-Terms User Interface (UI) supports including: browse terms **(A)**, suggest terms **(B)**, export terms **(C)**, and add new communities **(D)**.

The NIDM-Terms UI provides the following supportive functions: browse, search, edit, and export available terms and their properties. The “Browse Terms” function (Figure 2, Panel A) fetches the NIDM-Terms GitHub repository and displays the JSON-LD formatted files in a treeview format, including a tag for the term’s data type (e.g., concepts and data elements). Users are then able to filter through the available communities and terms based on the label of the term they’re interested in. We have developed additional functionality that allow users to suggest edits to the available terms and their properties, across the neuroimaging communities hosted on the UI. The UI will create a JSON-LD formatted dictionary with the user’s suggested edits to a specific term and using the edits as a query parameter string. Upon submission, a new browser tab will open a new Github pull request, with the edited term and its properties, to the NIDM-Terms repository allowing the user to use their login information to complete the pull request. The “Suggest new terms” function (Figure 2, Panel B) works in a similar manner to edit terms. Suggested terms will be formatted as a JSON-LD file. The JSON-LD file is then stringified and sent as a query parameter to the pull request to the NIDM-Terms repository; in case of any technical difficulties, the UI will submit a github issue to the NIDM-Terms repository with the suggested term describing the problem specifics while submitting the pull request. Upon the term’s approval by a community’s curator, a JSON-LD representation of the new terms will be added to the repository and a tree-view display of the new term will appear under the “Browse Terms” section of the UI. Note, each community has its own curators responsible for approving/interacting with users suggesting new terms and/or editing terms. In this way, each community has responsibility for their own terms. The “Export selected terms” function allows users to export terms, across communities, along with their properties, in several file formats: (1) A Markdown table for possible inclusion in community’s documentation (e.g., BIDS reference manual); (2) JSON; (3) JSON-LD; (4) CSV; (5) N-Quads (Figure 2, Panel C). Finally, the “Add a new community” function which allows for the addition of a new community to NIDM-Terms. Similar to “Suggest Terms,” the “Add a new community” functionality submits a pull request with the new community as a query parameter. Upon submitting new terms and communities to the NIDM-Terms GitHub repository, all new terms are validated using our SHACL Schema Validator (see section “2.3. SHACL validation”), consistent with the term properties described in (section “2.2. Properties”).

To further enhance our list of neuroimaging communities and support communities who may want complete control over their repository, we have provided instructions for cloning the NIDM-Terms UI and associated GitHub repository in order to host their own community in their name-space prior to merging them with the NIDM-Terms repository for broader community use. Together, these tools form a user-friendly interface allowing the neuroimaging community curators to interact with and reuse terminologies across communities, backed by a version control system.

### 3.2. Dataset annotation tools

In this project, we have created several tools to assist the neuroimaging community in annotating datasets using the terminology management tools we developed (see section “3.1. NIDM-Terms user interface”), consistent with our data element types and properties. This includes defining study-specific variables and their properties, as well as linking the variables to higher level concepts using the properties in Table 1. A rich set of annotations increases a dataset’s Findability and Reusability and can make publicly available datasets more FAIR by enabling scientists to efficiently discover datasets using concept-based queries.

To achieve this goal, we have built several annotation tools that allow scientists to efficiently and effectively annotate their study variables. We have built both command-line and graphical annotation tools. First, the “bidsmri2nidm” tool enables scientists to annotate BIDS structured datasets by iterating over the dataset and its variables contained in the “participants.tsv” file or other phenotypic files stored in the “phenotypes” directory through a command-line interface. A series of questions about each study variable will then be displayed on the screen allowing users to input specific properties describing those variables such as description, unit, minimum value, maximum value, etc. Additionally, the tool queries concepts from information sources such as InterLex and Cognitive Atlas for users to select the best matching concept to their study variable. The tool suggests concepts that are fuzzy-matched to the study variable name and provides a mechanism for users to refine such queries. Often when searching large information sources such as InterLex for concepts, users might find multiple concepts that could be applicable to the variable to be annotated. In our annotation tools, we initially present to the user the term deemed closest to the study variable amongst the list of concepts used for prior data annotations. For example, if annotating a study variable that stores the age of the participant, each data set provider should annotate this variable with the same “age” concept to increase consistent term usage. Our tools attempt to restrict the space of concepts by re-using concepts already used in data annotations from other users. In this way, we reduce the space to a single concept for “age.” The user can always broaden their search for concepts but this initial reduction in the search space helps to steer the user in selecting a concept that increases the potential for finding these data across studies. This reduction in search space is accomplished by the tool searching the NIDM-Terms github repository which maintains a list of concepts selected for annotations by users of the tool. To prevent duplicate choices for common study variables often used in queries (e.g., age, sex, handedness) the list of prior concepts is currently being manually curated. This is a place ripe for development using AI natural language processing techniques to keep the list of concepts relatively small and consistent.

After the annotation process is completed, the tool will export a JSON dictionary with the variables and their properties in addition to a NIDM-Experiment RDF document. This tool is a great addition to the neuroimaging community because it allows scientists to more easily add detailed and standard annotations to their BIDS structured datasets. In addition to “bidsmri2nidm,” we have also developed “csv2nidm,” which also allows for annotation of study variables however it uses tabular data [e.g., comma-separated values files (CSV) or tab–separated values files (TSV)] instead of requiring a complete BIDS datasets. Both of our command line interface tools, “bidsmri2nidm” and “csv2nidm” are open source and available with the PyNIDM (see text footnote 10) tools. To expand the use of our tools, we have additionally built a user-friendly web-based Graphical user interface version of csv2nidm^24^.

**FIGURE 3:**
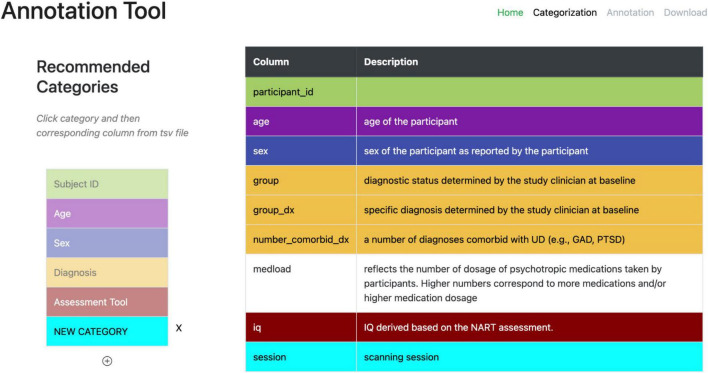
Stage 1 of the neurobagel annotator workflow. **Left:** a list of pre-defined categories (common data elements) is shown, each associated with a specific color. **Right:** the column names of a loaded demographic.tsv file with optional descriptions are displayed. The user now selects each category by clicking on it (e.g., “Diagnosis” in yellow), and then associates the category with each column that contains information about this category by clicking on it (e.g., “group,” “group_dx,” and “number_comorbid_dx”). An existing association is reflected by the column being highlighted in the color of the category.

**FIGURE 4:**
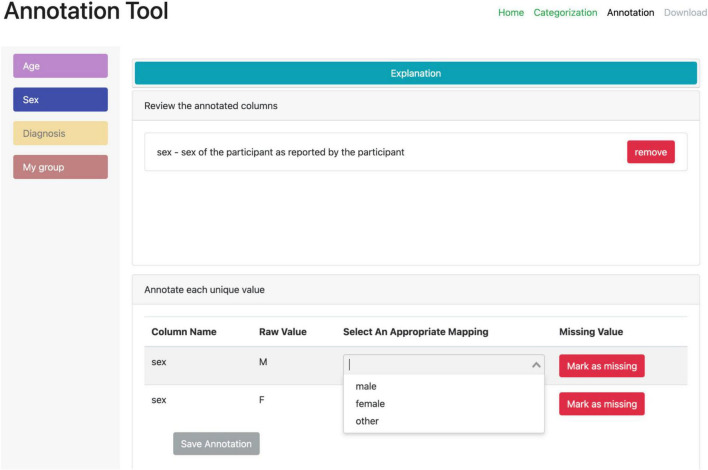
Stage 2 of the neurobagel annotator workflow. The user is now asked to annotate the values inside each column that has been linked to one of the predefined categories. **Left:** each category associated with at least one column is represented with a colored button. By clicking on each button, the user can annotate the values in the associated columns. **Right:** the annotation view for each category (here “Sex” in blue) contains specific elements such as an explanation (collapsed here), an overview of the associated column names (here “sex”), and an overview of the unique values in the associated column **(bottom)**. The user can map each unique value to a pre-defined list of controlled terms (here with a drop-down menu) or indicate that the value reflects a “missing value” (e.g., a data entry error or a truly missing response).

A second web-based annotation tool that has been developed by the ReproNim^25^ community is the Neurobagel^26^ annotation tool. This graphical annotation tool loads a tabular phenotypic file - for example a BIDS participants.tsv file–and then guides the user through two annotation stages. In the first stage (Figure 3), the user is presented with a number of pre-configured categories (i.e., CDEs), which have been previously agreed on across a number of dataset providers, and is asked to identify the columns of the loaded phenotypic file that contain information about each category (e.g., sex, or clinical diagnosis). To accomplish this step in the user interface (UI), the user first selects a category by clicking on the corresponding colored button, and then clicks on each column from the phenotypic file that she wants to associate with the category. An existing association between a column and a category is represented in the UI by highlighting the column name with the respective category color. In the second stage of the annotation process (Figure 4), the user is asked to annotate the values in each column that has been associated with a category (continuous values can be transformed into a standardized format). Each category has a predefined list of terms from a controlled vocabulary that a user has to choose from to annotate the values in their phenotypic file (from a list of common data elements). Constraining the annotation terms is a design choice to make the annotation process easier and to facilitate consistency across annotations at the expense of flexibility. However, the predefined categories will be configurable in the next version of the annotation tool to help communities choose the most appropriate set of terminologies. After completing the annotation, the neurobagel annotator creates a BIDS compatible data dictionary (JSON) file, that contains the additional semantic annotations as additional properties and can be converted to a NIDM file.

These annotation tools are beneficial for the neuroimaging community because they allow users to quickly and accurately annotate their study variables in a standardized way. This helps ensure that their data is consistently structured and more easily understood by other researchers working on similar projects and to facilitate cross-dataset queries. By integrating Interlex and Cognitive Atlas, it also allows scientists to quickly and easily match their variables to existing concepts, making it easier to formulate sophisticated scientific queries and to interpret their results.

### 3.3. Use case

**FIGURE 5:**
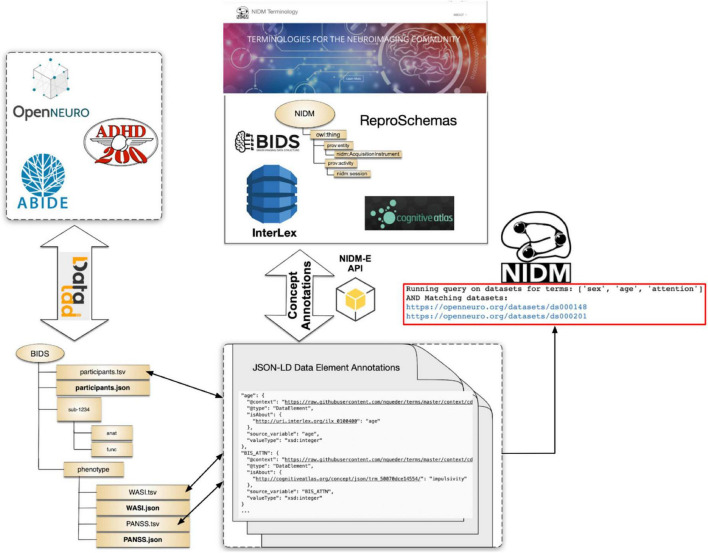
An illustration of the NIDM-Terms workflow.

To evaluate the developed tools and overall terminology management, annotation, and query workflows presented in this manuscript section, we focus on a specific use-case, that of querying across publicly available MRI-related neuroimaging data to identify potential cohorts of interest. For these tests we use publicly available projects contained in the OpenNeuro archive at the time our work began, the ABIDE^27^ dataset, and the ADHD200^28^ dataset, all of which are available from each dataset provider and were accessed using DataLad^29^ (Halchenko et al., 2021; Figure 5). Each of these datasets and the projects within the OpenNeuro archive are available in the BIDS format and generally contain MRI imaging data along with selected demographics and additional cognitive and/or behavioral assessments at varying levels of complexity. In addition, the selected datasets contain differing amounts of annotations. For ABIDE and ADHD200 datasets, full data dictionaries are available from the dataset providers; although, not in a readily parsable format (e.g., PDF format). For OpenNeuro projects, approximately 25% had annotations in the form of BIDS “sidecar” JSON files and the rest did not.

To prepare these reference datasets for query, given their varying levels of existing annotations and organizational form (e.g., BIDS containing phenotype data, BIDS for imaging data and phenotype data stored as separate tabular data files outside of BIDS), we used various NIDM-related tools. For the ABIDE study, each study site created their own BIDS dataset containing the imaging data. When we started our work, the phenotype data was stored separately for all sites as a CSV file. In later versions of the BIDS datasets, phenotype data was stored in the BIDS “participants.tsv” files. Although each study site collected the same phenotypic variables, it was often the case that the variable names were slightly different across sites in terms of the spelling, capitalization, and word-connection indicators such as spaces, dashes, or underscores. This inconsistency, even within a single study, demonstrates the difficulties users may have in trying to query across datasets simply using variable names. Further, there were no BIDS “sidecar” files included with any of the site’s BIDS datasets. To convert the ABIDE BIDS datasets into a NIDM document for query we used the following procedure:

• Download each ABIDE site’s BIDS dataset via Datalad.
• Manually convert the PDF-formatted data dictionary into a NIDM JSON-formatted data dictionary.
  ○ Add entries to JSON-formatted data dictionaries to accommodate all heterogeneity in variable naming across ABIDE sites.
  ○ Add high-level concept associations to the JSON-formatted data dictionary for selected variables using the isAbout property.
• For each ABIDE site.
  ○ Run PyNIDM tool “bidsmri2nidm” with a local path to the BIDS dataset.
  ○ Run PyNIDM tool “csv2nidm” with a local path to the phenotype CSV file, the JSON-formatted data dictionary, and the NIDM file created by the “bidsmri2nidm” step above.

The procedure above results in one NIDM document per site, containing both the imaging and phenotype metadata, along with the data dictionaries and concept annotations. In this procedure we created the JSON-formatted data dictionary and did concept associations manually since we already had a reference PDF document with variable definitions. Alternatively, one could use any of the annotation tools discussed in (section “3.2. Dataset annotation tools”).

To prepare the ADHD200 dataset for query we follow a similar procedure as for the ABIDE dataset except that the BIDS formatted dataset contained the imaging and phenotypic data. Similar to ABIDE, each study site’s BIDS data was stored separately and there was a variety of variable name heterogeneity. Here again, to improve the efficiency and account for the heterogeneity of variable names, we manually created the data dictionaries by transcribing the information from PDF-formatted documents. Similar to the ABIDE dataset, one could have used our annotation tools (from section “3.2. Dataset annotation tools”) as an alternative approach, which we think is far easier and less prone to transcription errors. Yet to be able to capture the heterogeneity of variable names across all the sites, we would have had to run ‘bidsmri2nidm‘ many times, once for each site, answering all the annotation questions about variables that have already been annotated but have slightly different names (e.g., one as a space whereas another site used an underscore). To complete this task at the scale we were working at, it was simpler for us to create a single data dictionary with all the variable name variations and provide this to the “bidsmri2nidm” tool. At the end of this procedure, we have a NIDM document per site containing both the imaging and phenotype data along with the data dictionaries and concept annotations.

Finally, we created NIDM documents for each dataset available in the OpenNeuro archive via DataLad at the time we performed these experiments. For the datasets in the OpenNeuro archive we used the following procedure:

• Download each OpenNeuro BIDS dataset via Datalad.
• Evaluate whether a data dictionary (JSON sidecar file) is available and export all variable names and properties to a Google spreadsheet along with project name and contact emails.
  ○ If data dictionary is present.
    ▪ Add concept annotations to the spreadsheet manually.
  ○ If data dictionary is not present.
    ▪ Evaluate variables for consistency with BIDS schema recommended data type, units, etc. (e.g., age variable suggested to be years, etc.).
    ▪ Ask dataset providers for clarity when needed.
    ▪ Add concept annotations to spreadsheet manually.
• Convert Google spreadsheet entries to BIDS JSON sidecar files for each project using our additions.
• Run PyNIDM tool ‘bidsmri2nidm‘ with a local path to the BIDS dataset and using our BIDS JSON “sidecar” files.

The procedure above resulted in the creation of a NIDM document for each OpenNeuro dataset available via Datalad at the time of initial query. For these datasets we used a different procedure from the ABIDE and ADHD200 NIDM conversions. Here we had to do the annotations in bulk for approximately 300 datasets while we developed, in parallel, the robust concept annotation capabilities of the ‘bidsmri2nidm‘ tool. To save time we decided to crowd-source the annotation activities amongst our NIDM-Terms team by using an export to a Google spreadsheet. As described previously, in practice for a smaller number of datasets, one could (and should) use any of the annotation tools provided with our work.

### 3.3. Concept-based queries

Now that each of our example datasets (i.e., OpenNeuro, ABIDE, and ADHD200) has been annotated using the methodologies presented here and a NIDM file representation created, we began testing concept-based integration queries. We created two Jupyter notebook query demonstrations available directly in the NIDM-Terms GitHub repository via Binder (see README - Demos^30^): (1) Using the JSON-LD version of our BIDS-compliant JSON “sidecar” files to query across OpenNeuro datasets; (2) Using the NIDM files across all three datasets to search by concept and neuroimaging type. We feel these demonstrations serve to show how a user can query across BIDS datasets using concepts without any backend database (example 1) and using NIDM files across three datasets facilitated through the ReproLake metadata database (example 2) supported by ReproNim (see text footnote 25). In example 2, we add the additional capability of querying for image type alongside concepts. Note, there are many additional pieces of metadata in the NIDM files that could be included, along with the ones shown here, in a production query interface.

With respect to example 1, the Jupyter notebook starts by pulling all the JSON-LD “sidecar” files for the OpenNeuro datasets from the NIDM-Terms GitHub repo. It then creates a dictionary of the concepts used in annotating those data by accessing the “isAbout” property in those JSON-LD files. Next, it uses ipywidgets^31^ to create a simple drop-down interface within the Jupyter notebook listing all the concepts available across all annotated OpenNeuro datasets. The user can then add concepts to a query list and perform AND-based or OR-based queries on the list. The notebook then returns a list of datasets in OpenNeuro that satisfy the query with links out to the OpenNeuro interface for the datasets. These queries are fairly efficient and require no additional database backend.

With respect to example 2, first the NIDM files, created here, for all studies (i.e., ABIDE, ADHD200, and OpenNeuro) were uploaded to ReproLake. ReproLake is a publicly available metadata archive developed on the StarDog^32^ platform. Although it is still in development, ReproLake will, in the near future, provide a metadata archive containing NIDM files describing many publicly available neuroimaging datasets. Because querying large RDF graphs across thousands of datasets is quite resource intensive, using a database to support these queries makes them more efficient. One could instead use a local metadata database to store and query these NIDM files by cloning the “Simple2_NIDM_Examples”^33^ repository and looking in the folder. Different from example 1, there are no JSON-LD or JSON files used in this demonstration. Here we use the NIDM files directly, served by ReproLake. The Jupyter notebook begins by performing a SPARQL query, sent to the ReproLake server, on the NIDM documents to retrieve the concepts via the “isAbout” predicate. It then queries the neuroimaging scan types from the NIDM documents by looking for data acquisition activities in the NIDM graphs that contain the “nidm:hadImageContrastType” predicate, a term that is part of the NIDM terminology (see section “2.2. Properties”). Next, similar to example 1, these concepts and contrast types get added to ipywidgets and the user can select criteria to query on. The tool then formulates a SPARQL query, presenting this query to the user for educational purposes, and sends the query to the ReproLake StarDog instance. Depending on the complexity of the query, the results can take a few seconds to many minutes (or longer) to complete. Because the ReproLake utility is still in development, no server-side optimizations have been done and limited server resources are available. As ReproNim continues to develop this resource, query response will improve.

## 4. Discussion

The dataset annotations and terminology management tools presented here have shown to be a useful and pragmatic approach to querying across datasets and linking datasets through mappings from dataset-specific variables and terms to broader concepts. Most of the tools and techniques presented here have been pragmatically-focused and developed, in part, to support building the ReproLake metadata database. We’ve tried to create models that are sufficiently expressive to capture important information needed to enable data reuse, while minimizing the burden on researchers. Thus far, through efforts connected to ReproNim and the overall NIDM work, we have found the minimal set of properties we’ve selected to be sufficient to find and reuse data, amongst the datasets we chose.

Through our query demonstrations and additional work with the ReproLake, concept annotations have been successful in helping us search across datasets. During our initial experiments, using the datasets described here and annotated by several individuals in our research team, we found that there was some ambiguity surrounding several similar concept choices. Even for simple variables such as age, sex, and handedness, there were multiple concepts that could be selected from the many available in large terminology management resources such as InterLex. To address this complexity, we’ve taken two main approaches, enabled by our choice to use RDF and JSON-LD: (1) constrain the search space for often-used concepts; (2) use RDF and linked-data capabilities to start connecting similar/equivalent terms in InterLex. Constraining the search space was accomplished using our NIDM-Terms GitHub repository to maintain a list of concepts selected for annotating previous datasets and to initially present those concepts to users of our tools, effectively giving them a single choice for age, sex, and handedness concepts. This procedure works well if the annotations are performed using our tools and curated term lists but does not address the problem when users are manually annotating data using term resources without guidance. The second approach, that of connecting similar/equivalent terms together within InterLex, has been an on-going project for many years and that project continues to make progress on that front. By connecting terms within InterLex using the RDF framework, one could perform equivalence mapping at query time via the SPARQL query language. Then, one could theoretically select concepts from InterLex without much concern for whether other dataset providers selected the same concept because the similarity and/or equivalency has already been modeled by the InterLex team and is used directly within the ReproLake query engine. This approach would satisfy those doing manual annotations but only when using InterLex. To make this approach scale, the research community should move toward using linked data methods across all metadata included with publicly available datasets. By creating a rich web of linked neuroimaging information, the overhead involved in database-dependent mediation services could be reduced and this linked terminology information would be available to any web resource. This is the promise of linked-data and we are seeing signs of this goal coming to fruition in the broader web, outside of neuroimaging-based scientific data.

Data-sharing requirements from funding agencies and journals, have done much to increase the amount of data available for reuse in the neuroimaging and other related communities over the last 10 years. The work presented here has been successful at providing a framework for annotating study variables in ways to make them more reusable by providing a formal (and minimal) list of properties and tools to support them in the context of the popular BIDS data structure. Further, the process of linking concepts to selected study variables has been successful at showing the promise of an integrated metadata search utility (i.e., ReproLake). Despite these advances, there is still much work to be done to realize a web of linked neuroimaging (neuroscience) data that is fully reusable and findable at scale and across studies. Through continued support from funding bodies and international informatics organizations such as the International Neuroinformatics Coordinating Facility (INCF),^34^ we expect the remaining barriers to slowly crumble such that data shared by any laboratory, globally, could be reused for the advancement of science.

## Data availability statement

Publicly available datasets were analyzed in this study. The annotated terms from those datasets and their properties can be found here: https://github.com/nidm-terms.

## Author contributions

All authors listed have made a substantial, direct, and intellectual contribution to the work, and approved it for publication.

## Footnotes

1
https://grants.nih.gov/grants/guide/notice-files/NOT-OD-21-013.html

2
https://coins.trendscenter.org/

3
https://www.xnat.org/

4
https://scitran.github.io/

5
https://openfmri.org/

6
http://mcin-cnim.ca/neuroimagingtechnologies/loris/

7
https://github.com/bids-standard/pybids

8
https://www.w3.org/TR/prov-overview/

9
http://nidm.nidash.org/

10
https://github.com/incf-nidash/PyNIDM

11
https://frictionlessdata.io

12
http://uri.interlex.org/ilx_0104886

13
https://www.w3.org/TR/prov-o

14
https://www.dicomstandard.org/

15
https://github.com/incf-nidash/nidm-experiment

16
https://incf-nidash.github.io/nidm-experiment/schema_menu.html

17
https://incf-nidash.github.io/nidm-experiment/

18
https://github.com/ReproNim/reproschema-py

19
https://scicrunch.org/scicrunch/interlex/dashboard

20
http://www.cognitiveatlas.org

21
https://nidm-terms.github.io/

22
https://github.com/NIDM-Terms/terms

23
https://json-ld.org/

24
https://incf-nidash.github.io/nidmterms-ui/?#/annotate

25
https://www.repronim.org/

26
https://annotate.neurobagel.org

27
http://fcon_1000.projects.nitrc.org/indi/abide/abide_I.html

28
http://fcon_1000.projects.nitrc.org/indi/adhd200/

29
https://www.datalad.org/

30
https://github.com/NIDM-Terms/terms/blob/master/README.md

31
https://ipywidgets.readthedocs.io/en/stable/

32
https://www.stardog.com/

33
https://github.com/dbkeator/simple2_NIDM_examples/tree/master/datasets.datalad.org

34
https://www.incf.org/
